# Cognitive Performance in Short Sleep Young Adults with Different Physical Activity Levels: A Cross-Sectional fNIRS Study

**DOI:** 10.3390/brainsci13020171

**Published:** 2023-01-19

**Authors:** Yanwei You, Jianxiu Liu, Dizhi Wang, Yingyao Fu, Ruidong Liu, Xindong Ma

**Affiliations:** 1 Division of Sports Science & Physical Education, Tsinghua University, Beijing 100084, China; 2 School of Social Sciences, Tsinghua University, Beijing 100084, China; 3 Vanke School of Public Health, Tsinghua University, Beijing 100084, China; 4 Beijing Jianhua Experimental Etown School, Beijing 100176, China; 5 Sports Coaching College, Beijing Sport University, Beijing 100091, China; 6 IDG/McGovern Institute for Brain Research, Tsinghua University, Beijing 100084, China

**Keywords:** Stroop test, cognitive performance, short sleep, fNIRS, physical activity

## Abstract

Short sleep is a common issue nowadays. The purpose of this study was to investigate prefrontal cortical hemodynamics by evaluating changes in concentrations of oxygenated hemoglobin (HbO) in cognitive tests among short-sleep young adults and to explore the relationship between sleep duration, physical activity level, and cognitive function in this specific population. A total of 46 participants (25 males and 21 females) were included in our study, and among them, the average sleep duration was 358 min/day. Stroop performance in the short sleep population was linked to higher levels cortical activation in distinct parts of the left middle frontal gyrus. This study found that moderate-to-vigorous physical activity (MVPA) was significantly associated with lower accuracy of incongruent Stroop test. The dose-response relationship between sleep duration and Stroop performance under different levels of light-intensity physical activity (LPA) and MVPA was further explored, and increasing sleep time for different PA level was associated with better Stroop performance. In summary, this present study provided neurobehavioral evidence between cortical hemodynamics and cognitive function in the short sleep population. Furthermore, our findings indicated that, in younger adults with short sleep, more MVPA was associated with worse cognitive performance. Short sleep young adults should increase sleep time, rather than more MVPA, to achieve better cognitive function.

## 1. Introduction

Sleep is essential during the whole life cycle, especially for those young adults, who are in a formative period of growth. Young adults undergo significant brain and body development during this critical period, and current findings suggest that a critical aspect of adolescent brain development is the remodeling of structural and functional connectivity, which supports functional specialization and cognitive function [1,2]. By referring the final report of National Sleep Foundation’s updated sleep duration recommendations, preschoolers are recommended to sleep for 10–13 h, school-aged children for 9–11 h, teenagers for 8–10 h, and young adults for 7–9 h [3,4]. Unfortunately, evidence indicated that many of them have access to far less sleep than they need. In fact, the National Sleep Foundation poll of 17–18 year-old teens reported that 75% obtained less than 8 h sleep per night [5]. This situation may be getting worse, and data conducted from one recent survey found that nearly 69% of high school students obtained seven or fewer hours of sleep per night [6].

It has been proved that short sleep degrades aspects of neurocognitive performance [5]. A number of neurocognitive fields, including cognitive functions, executive attention, working memory, and mental health, are particularly vulnerable to short sleep [7,8]. A mice study showed that short-term sleep deprivation could impair short-term learning memory [9], and long-term sleep deprivation could cause object recognition memory decline [10]. In human’s studies, functional metabolic and neurophysiological demonstrated that neural systems involved in executive function (i.e., prefrontal cortex) are more susceptible to sleep deprivation [11,12].

Functional near-infrared spectroscopy (fNIRS) is an emerging method to explore the oxygen change in the brain. As a noninvasive optical neuroimaging method, fNIRS can efficiently reflect the local changes in cerebral hemodynamics triggered by neural activities [13,14,15]. According to the neuronal oxygen metabolism theory, it can be explained that oxygen is used for energy production, resulting in a decrease in the concentration of oxygenated hemoglobin (HbO) accompanied by an increase in the concentration of deoxygenated hemoglobin (HbR) [16,17].

Most studies suggest regular physical activity (PA), as a window of opportunity, can promote not only physical health, but also mental health [18,19,20]. PA is a healthy lifestyle to improve cognition, attributed to its effect on several perspectives: shaping neural structure [21,22], secreting neurotrophins [23], and improving synaptic plasticity [24]. Additionally, regular exercise is beneficial to stress relief, sleep health, and executive functioning, which can lead to an overall improvement in cognitive performance [25,26,27]. However, there are still two points to be controversial: one is that whether PA is always effective in all population (including short sleep population in our study). Another is that the measurement of PA (i.e., subjective or objective means) can affect the results of the study finding.

To sum up, limited evidence has focused on the relationship between sleep duration, physical activity, and cognitive performance in short-sleep young adults, and little is known of the hemodynamics on the activation of the prefrontal cortex during cognitive tasks in this population. In view of the fact mentioned above, this cross-sectional study aims to answer the following three key questions: for the short sleep population, (i) which brain area is significantly activated in cognitive test from the perspective of hemodynamics; (ii) the association between physical activity level and cognitive performance; (iii) the relationship between sleep duration and cognitive performance under different levels of physical activity.

## 2. Materials and Methods

### 2.1. Participants and Study Design

A total of 61 healthy right-handed, young adults were initially recruited. Subsequently, they were asked to wear an ActiGraph GT3X+ accelerometer (ActiGraph LLC, Pensacola, FL, USA) to verify their sleep hours per day and physical activity status for 7 consecutive days. Three participants dropped out of the study without competing the Stroop test. 50 participants were identified with objective sleep data less than 7 h (420 min). 4 participants were identified with invalid physical activity data. Finally, 46 participants with eligible data were used for the final analysis.

None of the participants reported any history of chronic diseases. The study was approved by the academic ethics committee of Tsinghua University (IRB 20190091) and was in accordance with the Declaration of Helsinki (1964). All participants were informed about the study procedures and provided written informed consent to participate. In this cross-sectional research, participants were invited to visit our laboratory to assess the demographic information, wearing an accelerometer to examine sleep and regular physical activity rhythm, to conduct selected neuropsychological tests, and to assess their handedness and maximal isometric handgrip strength. Furthermore, fNIRS was used to record the cortical hemodynamics while the participants solved the Sternberg task. All tests were described below in more detail.

### 2.2. Cognitive Test Design

A computer-based version of the color-word matching Stroop task was applied to reflect cognitive performance. It consisted of word-name matching tasks (including neutral and incongruent conditions). To ensure equal visual content, the color words “green”, “red”, and “blue” were used. During tests, participants were required to press arrow buttons on the keyboard to identify the corresponding color of the ink rather than the actual meaning of the word, which was displayed in the center of the computer screen. Additionally, they were instructed to react as quickly and accurately as possible.

Referring to Ludyga’s study [28], our Stroop was set as six test blocks containing 36 trials, with 30 s resting periods between each block. Before the formal test, two training blocks were provided for practice. Neutral and incongruent test blocks were arranged with equal probability and followed a fully randomized order. The presenting time of color words was 250 ms, and it was asked for the reaction to be decided within a 1500 ms time window. In order to avoid habituation, the interstimulus interval was set randomly between 1000 and 1300 ms.

### 2.3. Physical Pattern Assessment

Previous research reported that subjective and objective measurement of sleep and physical activity could have disagreement on study findings [29], and objective strategy such as accelerometer measurement could have a complete representation on physical patterns. Hence, in this study, a multi-day accelerometer and ActiGraph GT3X+, (ActiGraph LLC, Pensacola, FL, USA) were applied to collect physical activity data. Participants wore accelerometer on the wrist of their non-dominant hand for 7 consecutive days, except during showering and water-based activities [30]. Referring to previous settings [31,32,33], the minimum minutes of the missing interval was defined as 60 min; the valid days were defined as more than 600 min of wearing; and the minimum number of valid days that the subject should have was defined as 3 days. According to classic criteria for cut points of physical activity level by counts per minute (CPM) [34], PA level was classified into light-intensity physical activity (LPA) and moderate-to-vigorous physical activity (MVPA).

### 2.4. Functional Near-Infrared Spectroscopy Data Acquisition

The 24-channel fNIRS equipment (Oxymon MkIII and Octamon, Artinis Medical Systems, Elst, The Netherlands) was used to record the hemodynamic concentration changes. The position of the probe was set according to the international 10–20 system, and a probe set of 24 channels (8 transmitters and 10 receivers, 3 cm distance) was placed on the prefrontal cortex of the brain (Figure S1). The sampling frequency setting of fNIRS was 10 Hz and wavelengths of emitted light was 756 nm and 853 nm [35] to measure changes in HbO and HbR concentrations in each channel. A higher activation of cortex can be reflected by increment of HbO and decrement in HbR [17,36]. It has been reported that HbO signals provided a higher signal-to-noise ratio and higher retest reliability than HbR signals [37]. Hence, in this study, HbO signals were employed as markers of regional cortical activity. Participants were asked to wear earphones to avoid noises from the environment, as well as to relax their mind and close their eyes before formal tests. Functional near-infrared spectroscopy (fNIRS) data were recorded during the Stroop tests. One example of study condition was shown in Figure S2. The fNIRS-signals were assigned from optodes to specific brain regions by the location decider and the Broadmann atlas [38]. Details of spatial registration were provided in Table S1.

### 2.5. Statistical Analysis

The raw data were extracted from the Artinis software (Artinis Medical Systems, Oxysoft v.3.2). After the fNIRS data were collected, the software program NIRS-SPM (statistical parameric mapping for near-infrared spectroscopy, v4-r1) was further used for hemodynamic signal processing. NIRS-SPM is a software package for statistical analysis of near infrared spectroscopy (NIRS) signals based on SPM5 (http://www.fil.ion.ucl.ac.uk/spm/, accessed on 21 November 2022). NIRS-SPM can not only provide the activation of oxygen, deoxygenation, and total hemoglobin in the cerebral cortex, but also enable super-resolution activation and localization. In order to filter out noise and artifacts caused by breathing and heartbeat pulsations, Mayer waves, or other instrumental noise, NIRS-SPM provided a wavelet MDL (minimum description length) method for detrending algorithm [39]. The collected signals were fitted with hemodynamic functions, and the β value of each condition was obtained and analyzed. The GLM can be expressed as the following equation [40]:

z (t) = β • *f* (t) + ε

Primarily, a hemodynamic response function (HRF) is embedded in the GLM to predict the activations in HbO signals by Stroop stimulation. In this equation, z (t) reflects the concentration changes of HbO. Meanwhile, *f* (t) represents the predicted stimulation-specific response during cognitive tasks, which is the convolution of a given HRF and stimulation-specific boxcar function. ε is the error term. Specifically, the β value is calculated after fitting the GLM, which is the estimated amplitude of ΔHbO and reflects the hemodynamic activations of specific channels. A low-pass filter was applied in the shape of the hemodynamic response function. In addition, channel residual covariance estimation was employed to consider the correlation of channel-wise least square residuals [35]. The calculated *p* values were all corrected by multiple comparisons, and the FDR (false discovery rates) method was used for correction.

R (R Project for Statistical Computing, Vienna, Austria, 4.0.3) and MATLAB (MathWorks, Inc., Nettick, MA, USA, 2019b) were used to analyze the achieved data. BrainNet Viewer toolbox (MATLAB-based software package) [41] was used to visualize the brain images, and “corrplot” (R-based software package) was used to analyze the correlation among the 24 channels. We examined the bivariate relationships between measures of activations of the prefrontal cortex and Stroop performance by the linear regression model, and gender as a confounding factor was adjusted in the model. Additionally, considering that the relationship between Stroop performance and physical activity or sleep hour might be non-linear, subgroups were conducted with trisecting PA levels (i.e., minor, moderate and extensive). A significance level of all comparisons was set as *p*-Value < 0.05.

## 3. Results

### 3.1. Demographic Characteristics

As shown in Table 1, among the 46 participants included in our final study, over a half of them were males (54%). The average age of them was 24 years old. The average weight and BMI were 64 kg and 22.1 kg/m^2^. Participants in this study conducted about 410 min LPA and 98 min MVPA per day, which reached the daily PA level recommended by WHO. For the Stroop test, the average accuracy of the congruent and incongruent test was 99.5% and 98.9%. In addition, the average reaction time of congruent and incongruent test was 63.5% and 68.8%, respectively.

### 3.2. Correlations between Prefrontal Cortex Activation and Stroop Performance

The relationship between prefrontal cortex activation detected by fNIRS channels with accuracy and reaction time was assessed by linear regression models (controlling for the influence of gender). We evaluated the cortex activation by HbO index in the total 24 channels. When it comes to accuracy, we observed no significant correlation between the level of HbO in all channels with congruent Stroop test accuracy, however, significant activation of left middle frontal gyrus was identified through channel 17 (Table 2) in incongruent Stroop. With regard to reaction time, there was a significant correlation between activation of the left middle frontal gyrus reflected by channel 23 (which was close to channel 17) and congruent Stroop test, and no significant relationship between cortex activation and reaction time of incongruent Stroop test was identified (Table S2).

**Figure 1:**
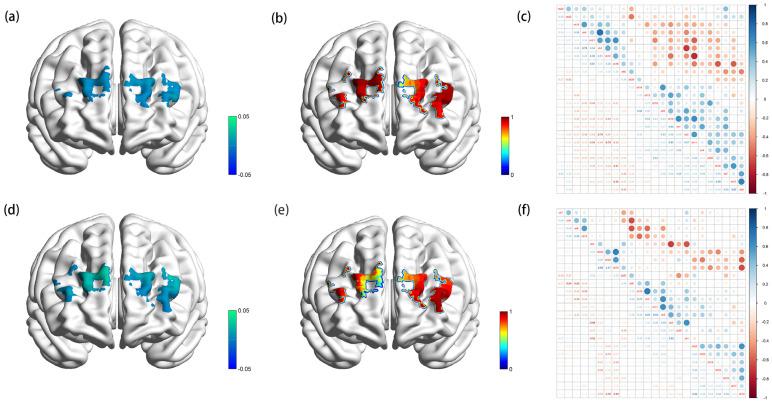
β-map reflected by oxygenated hemoglobin activation (**a**), p-map indicating the association between channel activation and accuracy (**b**), and Spearman correlation matrix of channel activation (**c**) of Stroop congruent test; β-map reflected by oxygenated hemoglobin activation (**d**), p-map indicating the association between channel activation and accuracy (**e**), and Spearman correlation matrix of channel activation (**f**) of Stroop incongruent test.

Figure 1 showed the visualized channel activation (a and c) as well as the *p*-value examining the significance between channel activation and Stroop accuracy in the prefrontal cortex in congruent (a, b and c) and incongruent test (d, e, and f). The correlations of channels activation (c and f) were evaluated by Spearman correlation analysis. As shown in Figure 1, for Stroop congruent test, the strongest correlation was found between channel 17 and channel 8 (r-Value = 0.67), which reflected the activation of the left middle frontal gyrus and right dorsolateral superior frontal gyrus. In the Stroop incongruent test, the strongest correlation was identified between channel 10 and channel 4 (r-Value = 0.71), indicating the activation of the right superior and middle frontal gyrus. In summary, better cognitive performance (faster reaction time and higher percentage of accuracy) in short sleep population was linked to higher levels cortical activation in distinct parts of the left middle frontal gyrus. Meanwhile, the right superior and middle frontal gyrus and right dorsolateral superior frontal gyrus also played roles in the executive process of the Stroop test.

### 3.3. Correlations between Sleep Duration and Physical Activity Levels with Stroop Performance

Linear regression models were constructed. In this study, sleep duration was categorized into light short sleep (6.5–7 h), mild short sleep (5.5–6.5 h), and severe short sleep (<5.5 h). Table 3 showed the relationship between sleep duration and Stroop test accuracy. Compared with light short sleep, mild and severe short sleep were associated with worse performance in congruent Stroop test accuracy (β (95%CI): −0.001(−0.008, 0.008), −0.005(−0.014, 0.004)) as well as incongruent Stroop test accuracy (β (95%CI): −0.005(−0.017, 0.006), −0.009(−0.022, 0.004)). Such negative associations were also found in the reaction time of congruent and incongruent Stroop test (Table S3). However, no statistical significance was found in such an association.

According to the guidelines of WHO and the volume of PA of participants in this study, light-intensity physical activity (LPA) was categorized into three level: minor (<6 h), moderate (6–8 h), and extensive (>8 h). Taking minor volume LPA as a reference, there was a positive association between LPA level and accuracy of congruent Stroop test (β (95%CI): 0.001(−0.009, 0.006), 0.001(−0.010, 0.011)), although the associations seemed to be not statistically significant. Moderate volume LPA was also detected to be positively associated with the accuracy of the incongruent Stroop test (0.008(−0.018, 0.002)), while extensive LPA showed the opposite trend (−0.006(−0.020, 0.008)). No significance was identified between LPA level and reaction time of congruent and incongruent Stroop test (Table S3).

Meanwhile, we also conducted the analysis of moderate-to-vigorous physical activity (MVPA) on Stroop performance. MVPA was grouped into trisecting levels: minor (<1 h), moderate (1–2 h), and extensive (>2 h). Moderate and extensive volume of MVPA was significantly associated with lower accuracy of incongruent Stroop test (β (95%CI): −0.011(−0.022, 0.001), −0.014(−0.028, −0.001)). However, no significant trends were identified between MVPA level and reaction time of congruent and incongruent Stroop test (Table S3).

**Figure 2:**
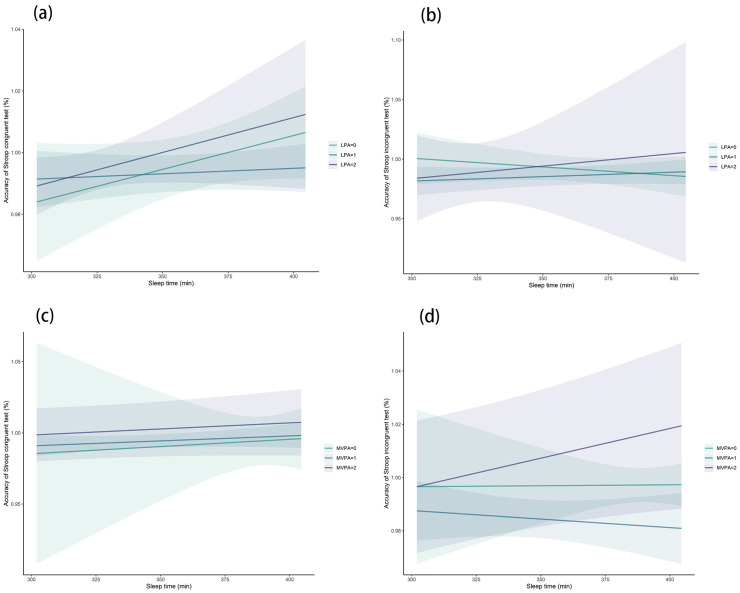
Association between sleep time and congruent (**a**) and incongruent (**b**) Stroop accuracy under different levels of light physical activity; association between sleep time and congruent (**c**) and incongruent (**d**) Stroop accuracy under different levels of moderate-to-vigorous physical activity.

We further investigated the dose-response relationship between sleep duration and Stroop performance under different levels of LPA and MVPA. From the perspective of LPA, whether LPA was minor to extensive, increasing the sleep hour for short sleep population was associated with better accuracy in congruent Stroop test (Figure 2a). This finding was also verified in moderate and extensive LPA group in the incongruent Stroop test, while minor LPA group showed a downward trend with the increment of sleep duration and test accuracy (Figure 2b). For different MVPA group, no significant trend was found with the increment of sleep hour and accuracy of congruent Stroop test (Figure 2c), while increasing sleep in extensive MVPA group was associated with higher accuracy in the incongruent Stroop test (Figure 2d).

## 4. Discussion

This current study explored the relationship between sleep duration, physical activity, cognitive performance, and cortical hemodynamics. We assessed physical patterns via a multi-day accelerometer (ActiGraph GT3X+) and evaluated prefrontal cortical hemodynamics during a classic cognitive test (Stroop) using fNIRS.

According to our results, the middle frontal gyrus made significant contributions to the Stroop performance in the short-sleep population. A total sleep deprivation study detected that an enhanced effective connectivity between the middle frontal gyrus and the parietal lobe in the task state [42]. One previous study using fNIRS to investigate the prefrontal cortical responses to deception under different motivations also verified this finding that the executive function test led to greater neural activation in the left middle frontal gyrus [43]. Our data were in agreement with another study that reported elevated cortical responses found in the middle frontal gyrus in background noise [44], which indicated the role of the middle frontal gyrus activation in the anti-interference task. In addition, this study also found the activation correlation between the right superior and middle frontal gyrus and right dorsolateral superior frontal gyrus, identifying the brain region-specific connections during the Stroop test. A prior study reported that Stroop interference-related activation was significantly enhanced in the dorsolateral prefrontal cortex, and acute moderate exercise could significantly activate this region to improve cognitive performance [45]. A similar study also found that acute high-intensity exercise might improve Stroop performance by shortening the Stroop test response time by evoking cortical activation on the left-dorsal-lateral prefrontal cortex. Although our data do not reflect these findings, they do complement the existing literature that both middle frontal gyrus and dorsolateral superior frontal gyrus activation played essential roles in cognitive performance.

We next investigated the relationship between physical activity and Stroop performance in this short-sleep population in a dosage-dependent manner. Our study found that moderate and extensive volume of MVPA was significantly associated with worse performance in the Stroop test. At a glance, these findings seem to contradict those of exercise’s benefits [46]. However, in consideration of the fact short sleep itself could affect the ability and motivation of exercise, it made sense that exercise intensity could be an important factor to account for cognitive performance. From mechanism, although sleep and physical exercise was regulated by completely different physiological pathways, there was growing evidence for crucial relationships between these two behaviors. Physical exercise was recognized as an arousal-provoking activity [47]. It has been suggested that acute exercise arose activation of the right frontal cortex [48], and exercise was an additional stressor to negatively impact reaction time and attentional lapses for the short-sleep population [49]. Additionally, a high volume of MVPA could bring more fatigue in short sleep population, which also led to a negative association between MVPA level and Stroop performance. Based on the above findings, our study further examined sleep duration and cognitive function under different levels of PA. Overall, it was suggested that whether performing from low-to-high volume of PA, increasing the sleep time was associated with better Stroop performance.

This cross-sectional study had several strengths as well as limitations, which should be discussed. In terms of strengths, to the best of our knowledge, this was the first study to purposefully investigate the association of hemodynamic activation and Stroop performance in specific short-sleep young adults by fNIRS. Objective measurement of accelerometers was applied in this study to evaluate the sleep duration and physical activity level, which was more accurate than the self-reported questionnaire. Moreover, another strength of this study was the analysis of the association between physical activity level and cognitive performance in this targeted population. Limited studies paid attention to the physical activity level and cognitive function in short-sleep person. By focusing on short-sleep groups, we provided a valuable extension of physical activity guidelines to the existing literature and opened the door to examine the bidirectional relationship between physical activity status and cognitive function, which showed exercise was not always effective. Thirdly, the relationships between sleep duration and cognitive performance under different levels of physical activity in the short-sleep population were assessed in a dose-response way, which further emphasized the importance of increasing sleep time rather than enhancing physical activity to improve cognitive performance.

Despite notable strengths, some limitations should be noted. Firstly, only the prefrontal cortex was selected as the region of interest. It was possible that other brain regions which the probe set did not cover were involved in the cognitive performance. Secondly, we only controlled gender as confounding factors for analysis, while short-sleep and physical activity status was influenced by multiple variables, such as individual social-demographic differences and other psychological or physiological factors. Future studies should use advanced brain image techniques and longitudinal designs with larger sample sizes to investigate individual differences to further confirm the cortex activation of short-sleep population. Last but not least, due to the cross-sectional design, we cannot deduce the causal inference of the relationship between sleep and physical activity level with cognitive performance. Our findings warranted further randomized controlled trials and biological studies to verify.

## 5. Conclusions

In summary, for the first time, our study explored, with objective measurement of sleep and physical activity level, the probable mechanism of cortex activation during the cognitive test of short-sleep young adults. We found that the left middle frontal gyrus made significant contributions to the Stroop performance. Meanwhile, the right superior and middle frontal gyrus, as well as the right dorsolateral superior frontal gyrus, also played roles in the executive process. Moreover, the associations between physical activity and sleep duration with cognitive performance were analyzed. A large volume of MVPA was significantly associated with worse performance in a cognitive test. According to our results, not all people were encouraged to engage in more MVPA, especially in short sleep conditions. We suggested that, in the short-sleep population, maintaining a balance between sleep and physical activity was crucial to cognitive functioning.

## Figures and Tables

**Table 1:** Demographic information of study participants.

Parameters	Mean + SD/N (%)
Gender	
Male	25 (54.348%)
Female	21 (45.652%)
Age (year)	23.717 ± 5.572
BMI (kg/m^2^)	22.080 ± 3.209
Weight (kg)	64.152 ± 12.822
LPA (min/day)	410.288 ± 85.501
MVPA (min/day)	97.837 ± 36.143
Sleep duration (min/day)	358.587 ± 34.091
Accuracy (congruent Stroop) (%)	0.995 ± 0.010
Accuracy (incongruent Stroop) (%)	0.989 ± 0.014
Reaction time (congruent Stroop) (s)	0.635 ± 0.055
Reaction time (incongruent Stroop) (s)	0.688 ± 0.071

Notes: BMI = body mass index, LPA = light physical activity, MVPA = moderate-to-vigorous physical activity.

**Table 2:** The associations between the regional cortical changes in the concentrations of oxygenated hemoglobin (HbO) with congruent and incongruent Stroop test accuracy.

	Accuracy of Congruent Stroop Test		Accuracy of Incongruent Stroop Test	
	β (95%CI)	*p*-Value ^	β (95%CI)	*p*-Value ^
Channel 1	−0.001(−0.006, 0.004)	0.976	0.002(−0.004, 0.009)	0.971
Channel 2	0.005(−0.002, 0.012)	0.827	−0.004(−0.014, 0.006)	0.971
Channel 3	−0.009(−0.022, 0.005)	0.827	0.006(−0.012, 0.024)	0.971
Channel 4	−0.001(−0.004, 0.003)	0.976	0.001(−0.004, 0.005)	0.976
Channel 5	0.003(−0.003, 0.009)	0.971	0.009(0.001, 0.016)	0.288
Channel 6	−0.002(−0.021, 0.016)	0.976	0.001(−0.024, 0.025)	0.976
Channel 7	0.001(−0.007, 0.008)	0.976	0.001(−0.009, 0.011)	0.976
Channel 8	−0.001(−0.007, 0.005)	0.976	−0.005(−0.013, 0.003)	0.827
Channel 9	−0.006(−0.016, 0.005)	0.864	−0.001(−0.015, 0.013)	0.976
Channel 10	0.002(−0.004, 0.008)	0.971	−0.001(−0.009, 0.007)	0.976
Channel 11	0.001(−0.007, 0.007)	0.976	−0.01(−0.019, −0.002)	0.288
Channel 12	0.005(−0.015, 0.025)	0.976	0.031(0.006, 0.056)	0.288
Channel 13	0.001(−0.005, 0.006)	0.976	0.004(−0.003, 0.012)	0.851
Channel 14	0.006(0.000, 0.012)	0.605	0.007(−0.002, 0.016)	0.713
Channel 15	−0.015(−0.042, 0.012)	0.851	−0.01(−0.047, 0.026)	0.976
Channel 16	0.002(−0.007, 0.010)	0.976	−0.009(−0.020, 0.002)	0.713
Channel 17	−0.004(− 0.010,0.002)	0.827	−0.012(−0.020, −0.005)	0.048 *
Channel 18	0.006(−0.021, 0.034)	0.976	0.025(−0.011, 0.061)	0.827
Channel 19	0.001(−0.004, 0.004)	0.976	0.001(−0.005, 0.005)	0.976
Channel 20	0.002(−0.004, 0.008)	0.971	−0.002(−0.009, 0.006)	0.976
Channel 21	−0.006(−0.018, 0.006)	0.864	0.001(−0.015, 0.018)	0.976
Channel 22	−0.003(−0.011, 0.006)	0.971	0.007(−0.004, 0.018)	0.827
Channel 23	−0.001(−0.007, 0.004)	0.976	−0.001(−0.009, 0.007)	0.976
Channel 24	0.013(−0.013, 0.040)	0.864	−0.011(−0.047, 0.024)	0.971

Notes: LPA = light physical activity, MVPA = moderate-to-vigorous physical activity, CI = confidence intervals, ^ *p*-Value was adjusted by FDR correction, * Significant with *p* < 0.05.

**Table 3:** The associations between sleep and physical activity with congruent and incongruent Stroop test accuracy.

	Accuracy of Congruent Stroop Test		Accuracy of Incongruent Stroop Test	
	β (95%CI)	*p*-Value	β (95%CI)	*p*-Value
**Sleep**				
Light short sleep	Reference		Reference	
Mild short sleep	−0.001(−0.008, 0.008)	0.956	−0.005(−0.017, 0.006)	0.337
Severe short sleep	−0.005(−0.014, 0.004)	0.231	−0.009(−0.022, 0.004)	0.172
**LPA**				
Minor	Reference		Reference	
Moderate	0.001(−0.009, 0.006)	0.774	0.008(−0.018, 0.002)	0.132
Extensive	0.001(−0.010, 0.011)	0.911	−0.006(−0.020, 0.008)	0.388
**MVPA**				
Minor	Reference		Reference	
Moderate	0.002(−0.007, 0.010)	0.695	−0.011(−0.022, 0.001)	0.046 *
Extensive	0.005(−0.005, 0.015)	0.358	−0.014(−0.028, −0.001)	0.039 *

Notes: LPA = light physical activity, MVPA = moderate-to-vigorous physical activity, CI = confidence intervals, * Significant with *p* < 0.05.

## Data Availability

The data presented in this study are available on request from the corresponding author.

## Supplementary Materials

The following supporting information can be downloaded at: https://www.mdpi.com/article/10.3390/brainsci13020171/s1, Supplement Figure S1 (Figure S1): Positions of fNIRS probe set. Numbers marked with yellow square background represents for the position of the 24 channels; Supplement Figure S2 (Figure S2). Experimental environment and examples of Stroop and fNIRS tests; Supplement Table S1 (Table S1). The MNI coordinates and anatomical labels corresponding to the 24 measurement channels; Supplement Table S2 (Table S2). The associations between the regional cortical changes in the concentrations of oxygenated hemoglobin (HbO) with congruent and incongruent Stroop reaction time; Supplement Table S3 (Table S3). The associations between sleep and physical activity with congruent and incongruent Stroop test reaction time.
