# Supplementary material for: Cognitive Performance in Short Sleep Young Adults with Different Physical Activity Levels: A Cross-Sectional fNIRS Study
Source: Brain Sci. 2023 Jan 19;13(2):171. doi: 10.3390/brainsci13020171 (PMC9954673; doi:10.3390/brainsci13020171)
Supplement: Supplementary file 1 [file brainsci-13-00171-s001.zip › brainsci-2143249-supplementary.docx]

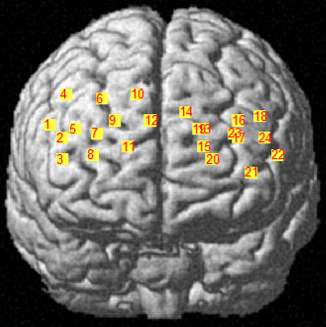


**Supplement Figure 1 (Figure S1).** Positions of fNIRS probe set. Numbers marked with yellow square background represents for the position of the 24 channels.


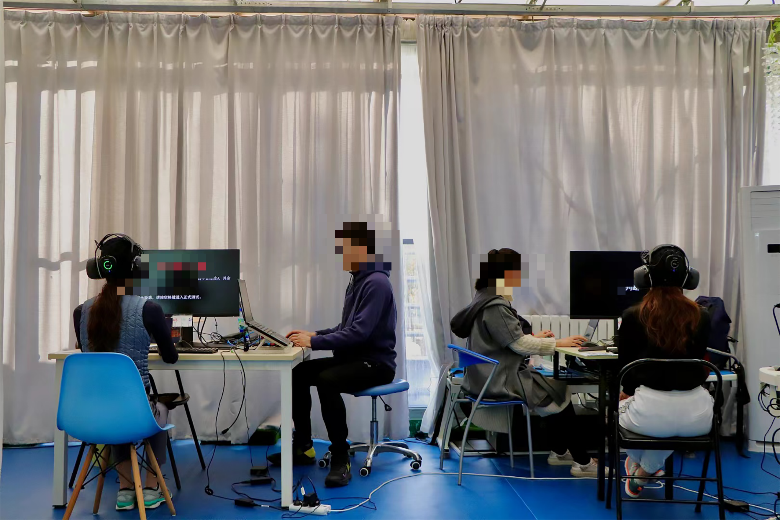


**Supplement Figure 2 (Figure S2)**. Experimental environment and examples of Stroop and fNIRS tests

**Supplement Table 1 (Table S1)**. The MNI coordinates and anatomical labels corresponding to the 24 measurement channels.

|  | MNI | | | **Brain Regions (Percentage of Overlap)** |
| --- | --- | --- | --- | --- |
|  | x | *y* | z |  |
| Channel 1 | 52 | 38 | 28 | Middle Frontal Gyrus (0.60) |
| Channel 2 | 46 | 51 | 22 | Middle Frontal Gyrus (1) |
| Channel 3 | 47 | 54 | 13 | Middle Frontal Gyrus (1) |
| Channel 4 | 44 | 36 | 42 | Middle Frontal Gyrus (1) |
| Channel 5 | 40 | 54 | 26 | Middle Frontal Gyrus (1) |
| Channel 6 | 27 | 52 | 40 | Dorsolateral Superior Frontal Gyrus (0.61) |
| Channel 7 | 31 | 63 | 23 | Middle Frontal Gyrus (0.57) |
| Channel 8 | 32 | 66 | 15 | Dorsolateral Superior Frontal Gyrus (0.75) |
| Channel 9 | 22 | 63 | 30 | Dorsolateral Superior Frontal Gyrus (0.65) |
| Channel 10 | 13 | 57 | 42 | Superior and Middle Frontal Gyrus (0.56) |
| Channel 11 | 16 | 71 | 19 | Dorsolateral Superior Frontal Gyrus (0.53) |
| Channel 12 | 6 | 65 | 31 | Superior and Middle Frontal Gyrus (0.58) |
| Channel 13 | -17 | 66 | 26 | Dorsolateral Superior Frontal Gyrus (0.84) |
| Channel 14 | -9 | 64 | 33 | Superior and Middle Frontal Gyrus (0.62) |
| Channel 15 | -17 | 70 | 18 | Dorsolateral Superior Frontal Gyrus (0.95) |
| Channel 16 | -33 | 54 | 30 | Middle Frontal Gyrus (0.89) |
| Channel 17 | -35 | 59 | 23 | Middle Frontal Gyrus (0.80) |
| Channel 18 | -45 | 41 | 32 | Middle Frontal Gyrus (0.88) |
| Channel 19 | -16 | 66 | 26 | Dorsolateral Superior Frontal Gyrus (0.75) |
| Channel 20 | -23 | 70 | 13 | Dorsolateral Superior Frontal Gyrus (0.98) |
| Channel 21 | -41 | 59 | 6 | Middle Frontal Gyrus (0.75) |
| Channel 22 | -52 | 41 | 14 | Superior and Middle Frontal Gyrus (0.81) |
| Channel 23 | -31 | 60 | 24 | Middle Frontal Gyrus (0.63) |
| Channel 24 | -46 | 47 | 21 | Middle Frontal Gyrus (0.79) |

**Supplement Table 2 (Table S2).** The associations between the regional cortical changes in the concentrations of oxygenated hemoglobin (HbO) with congruent and incongruent Stroop reaction time.

|  | Reaction time (congruent Stroop) (s) | | Reaction time (incongruent Stroop) (s) | |
| --- | --- | --- | --- | --- |
|  | β (95%CI) | *P-value^^^* | β (95%CI) | *P-value^^^* |
| Channel 1 | -0.013(-0.041,0.015) | 0.859 | 0.001(-0.037,0.038) | 0.984 |
| Channel 2 | 0.012(-0.030,0.054) | 0.957 | 0.046(-0.008,0.099) | 0.485 |
| Channel 3 | -0.005(-0.082,0.072) | 0.984 | -0.018(-0.118,0.082) | 0.957 |
| Channel 4 | -0.013(-0.032,0.006) | 0.654 | -0.002(-0.027,0.023) | 0.974 |
| Channel 5 | 0.012(-0.022,0.045) | 0.957 | 0.015(-0.029,0.058) | 0.957 |
| Channel 6 | 0.034(-0.069,0.136) | 0.957 | -0.015(-0.150,0.120) | 0.974 |
| Channel 7 | 0.001(-0.041,0.042) | 0.984 | 0.014(-0.040,0.068) | 0.957 |
| Channel 8 | -0.018(-0.053,0.017) | 0.816 | -0.004(-0.051,0.042) | 0.974 |
| Channel 9 | -0.017(-0.077,0.043) | 0.957 | -0.021(-0.098,0.057) | 0.957 |
| Channel 10 | 0.003(-0.029,0.036) | 0.974 | 0.032(-0.010,0.073) | 0.563 |
| Channel 11 | 0.007(-0.032,0.046) | 0.957 | 0.032(-0.018,0.082) | 0.703 |
| Channel 12 | 0.003(-0.110,0.116) | 0.984 | -0.026(-0.173,0.121) | 0.957 |
| Channel 13 | 0.016(-0.015,0.047) | 0.816 | 0.036(-0.003,0.076) | 0.450 |
| Channel 14 | 0.017(-0.020,0.054) | 0.859 | 0.016(-0.032,0.065) | 0.957 |
| Channel 15 | -0.023(-0.175,0.128) | 0.959 | -0.113(-0.308,0.081) | 0.768 |
| Channel 16 | -0.034(-0.080,0.012) | 0.576 | 0.001(-0.061,0.062) | 0.984 |
| Channel 17 | -0.027(-0.060,0.006) | 0.499 | -0.014(-0.058,0.031) | 0.957 |
| Channel 18 | 0.184( 0.042,0.326) | 0.168 | 0.113(-0.085,0.310) | 0.768 |
| Channel 19 | -0.005(-0.027,0.016) | 0.957 | -0.005(-0.033,0.022) | 0.957 |
| Channel 20 | -0.01(-0.041,0.021) | 0.957 | -0.001 (-0.041,0.040) | 0.984 |
| Channel 21 | 0.013(-0.054,0.080) | 0.957 | -0.015(-0.103,0.073) | 0.957 |
| Channel 22 | -0.047(-0.091,-0.003) | 0.304 | -0.053(-0.111,0.005) | 0.450 |
| Channel 23 | -0.049(-0.078,-0.021) | 0.048* | -0.056(-0.095,-0.017) | 0.144 |
| Channel 24 | 0.176( 0.038,0.314) | 0.168 | 0.196( 0.012,0.381) | 0.304 |

Notes: LPA = light physical activity, MVPA = moderate-to-vigorous physical activity, CI = confidence intervals, ^P-value was adjusted by FDR correction, * Significant with p < 0.05.

**Supplement Table 3 (Table S3).** The associations between sleep and physical activity with congruent and incongruent Stroop test reaction time.

|  | Reaction time (congruent Stroop) | | Reaction time (incongruent Stroop) | |
| --- | --- | --- | --- | --- |
|  | β (95%CI) | *P-value* | β (95%CI) | *P-value* |
| **Sleep** |  |  |  |  |
| Light short sleep | Reference |  | Reference |  |
| Mild short sleep | -0.02(-0.064,0.023) | 0.348 | -0.008(-0.066,0.050) | 0.782 |
| Severe short sleep | -0.046(-0.096,0.004) | 0.068 | -0.034(-0.101,0.032) | 0.301 |
| **LPA** |  |  |  |  |
| Minor | Reference |  | Reference |  |
| Moderate | -0.003(-0.045,0.039) | 0.890 | -0.026(-0.079,0.028) | 0.346 |
| Extensive | -0.005(-0.061,0.052) | 0.862 | -0.019(-0.092,0.054) | 0.608 |
| **MVPA** |  |  |  |  |
| Minor | Reference |  | Reference |  |
| Moderate | -0.008(-0.054,0.038) | 0.718 | -0.019(-0.079,0.041) | 0.535 |
| Extensive | -0.017(-0.073,0.040) | 0.555 | -0.026(-0.099,0.047) | 0.473 |

Notes: LPA = light physical activity, MVPA = moderate-to-vigorous physical activity, CI = confidence intervals.
